# Lower extremity deformity and its risk factors in patients with solitary osteochondromas

**DOI:** 10.1186/s13018-024-04908-4

**Published:** 2024-07-19

**Authors:** Seungtak Oh, Seung Hyun Won, Woo Sub Kim, Moon Seok Park, Ki Hyuk Sung

**Affiliations:** 1 Department of Orthopedic Surgery, Seoul National University College of Medicine, Seoul National University Bundang Hospital, 82 Gumi-ro 173 Beon-gil, Bundang-Gu, Sungnam, Gyeonggi 13620 Korea; 2 Division of Statistics, Medical Research Collaborating Center, Seoul National University Bundang Hospital, Gyeonggi, Korea; 3 Department of Orthopedic Surgery, Myongji Hospital, Hanyang University College of Medicine, Gyeonggi, Korea

**Keywords:** Solitary, Osteochondroma, Deformity, Risk factor

## Abstract

**Background:**

This study aimed to demonstrate the occurrence of lower extremity deformities and their risk factors in patients with solitary osteochondromas.

**Methods:**

We retrospectively reviewed consecutive patients with solitary osteochondromas around the knee. The laterality (left or right), involved bone (femur or tibia), tumor type (pedunculated or sessile), and direction (medial or lateral) were examined. The whole limb length (WLL), mechanical lateral distal femoral angle (mLDFA), and medial proximal tibial angle (MPTA) were measured using teleroentgenogram. Lower limb deformity was defined as a difference of more than 5° in mLDFA or MPTA in both lower extremities or a difference in WLL of more than 1 cm. Patients were divided into two groups, with deformity and without deformity.

**Results:**

Lower extremity deformities were observed in 8 of 83 patients. Significant difference in the type of osteochondroma (*p* = 0.004) between the groups was observed. Differences in sex, age, laterality, involved bone, direction, and distance from the physis to the osteochondroma between groups were not statistically significant. The sessile type of osteochondroma was a risk factor for lower limb deformity with an odds ratio of 24.0 according to Firth’s logistic regression analysis.

**Conclusion:**

In our cohort with solitary osteochondroma, lower limb deformities were observed in 8 (9.6%) out of the 83 patients and these were significantly associated with sessile-type tumors. Therefore, patients with sessile-type solitary osteochondroma around the knee require careful surveillance of lower limb alignment with whole leg teleroentgenogram.

## Introduction

An osteochondroma is a bony protrusion from the bone’s outer surface covered with cartilage. It is the most common bone tumor, accounting for 20–50% of benign bone tumors and 9% of all tumors. Clinical manifestations include pain, bony deformities, compression of the surrounding tissues, and vascular or neurological compromise [1]. Osteochondromas can be solitary or multiple, which is known as hereditary multiple exostoses (HME).

HME is an autosomal dominant skeletal disorder characterized by the growth of multiple osteochondromas at various sites, particularly the metaphyses of long bones. Several studies have investigated skeletal dysplasia in patients with HME. For example, Clement demonstrated that nine of 10 patients with HME had exostoses around the knee, 20% had a valgus knee, 16% had a fixed flexion deformity, and 8% had limb length discrepancies [2]. However, little is known about the deformities in patients with solitary osteochondroma.

Several cases of solitary osteochondromas with lower extremity deformities have been reported at our institution. We found that there are only a few studies on deformities of the lower extremities in solitary osteochondromas. Solitary osteochondromas have been shown to have little to do with the deformity [1, 3–5]. 

Therefore, the purpose of this study was to demonstrate that solitary osteochondroma can cause lower limb deformities. The risk factors for lower-extremity deformity in patients with solitary osteochondroma were analyzed. We hypothesized that the distance from the mass to the physis and the type of osteochondroma might influence the occurrence of lower limb deformities.

## Methods

The institutional review board of our hospital approved this study, and the requirement for informed consent was waived due to this study’s retrospective design.

We retrospectively reviewed consecutive patients aged < 30 years with osteochondroma between January 2006 and February 2023. We included patients with solitary osteochondromas around the knee joint. The exclusion criteria were as follows: (1) patients who had multiple osteochondromas located in different bones, and (2) patients who had osteochondroma located in anterior or posterior portion of the bone because this study focused on the coronal angular deformity.

Information regarding patients’ age, sex, date of initial diagnosis, and treatment was obtained from a review of medical records.

### Radiographic measurements

To assess coronal angular deformity, standing anteroposterior long-cassette radiographs of the lower extremity (teleroentgenogram) were obtained at a source-to-image distance of 200 cm and set to 50 kVp and 5 mAs, with the patella facing forward. The procedure involved single X-ray exposure of both lower limbs with an X-ray beam at the center of the knee. All measurements were performed using a PACS software package (Infinitt, Seoul, South Korea).

**Fig. 1:**
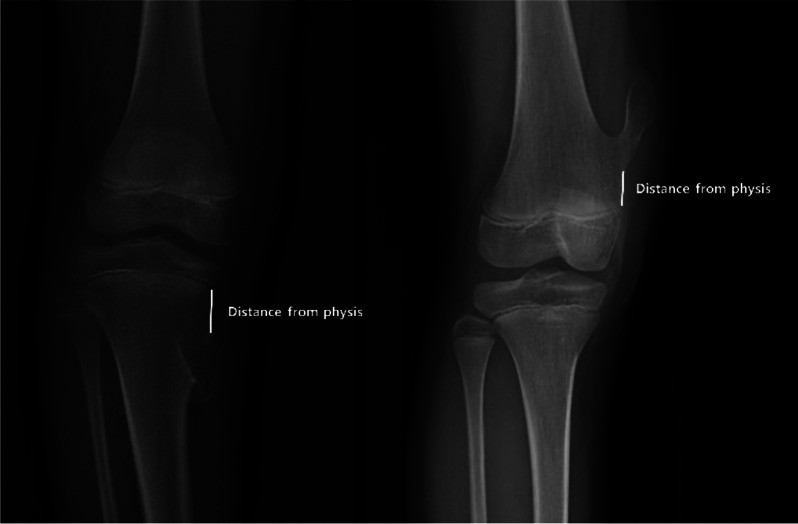
The distance between the tumor and the adjacent physeal plate for sessile and pedunculated tumors

**Fig. 2:**
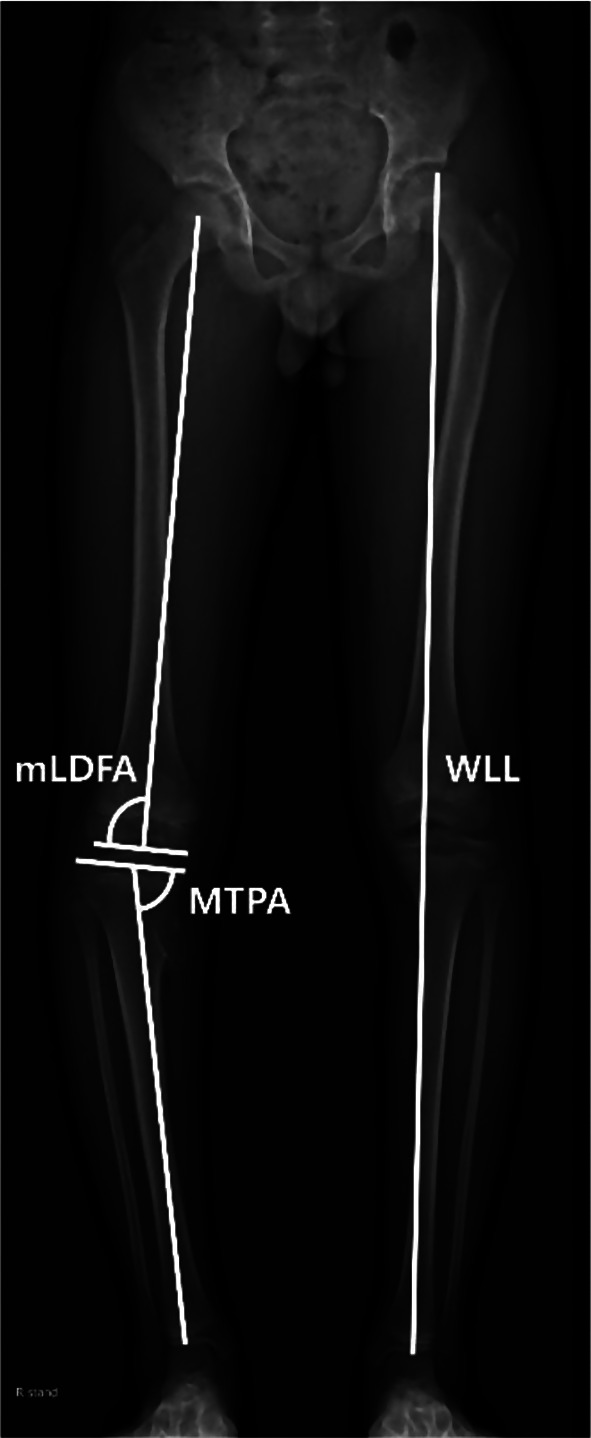
The mechanical lateral distal femoral angle (mLDFA) was defined as the angle formed by the line connecting the center of the femoral head and the center of the distal femoral epiphysis, and the knee joint line of the femur. The mechanical medial proximal tibial angle (mMPTA) was defined as the angle formed by the line connecting the center of the proximal tibial epiphysis and the center of the talar dome, and the knee joint line of the tibia. Whole limb length (WLL) was defined as the length from the top of the femoral head to the center of the tibial plafond

The laterality (left or right), bone involved (femur or tibia), type (pedunculated or sessile), and direction (medial or lateral) of the osteochondroma were examined. The distance between the osteochondroma and the adjacent physeal plate was also assessed (Fig. 1). Mechanical lateral distal femoral angle (mLDFA), medial proximal tibial angle (MPTA), and whole limb length (WLL) were measured. The mLDFA is defined as the lateral angle formed between the mechanical axis of the femur and a line drawn through the knee joint line of the femur in the frontal plane. The MPTA was defined as the medial angle between the tibial mechanical axis and the line drawn through the tibial knee joint in the frontal plane [6]. WLL was defined as the length from the top of the femoral head to the center of the tibial plafond (Fig. 2) [7]. 

Before the main measurement, inter-observer reliability for radiographic measurements was assessed. Two orthopedic surgeons (KHS and STO) independently performed radiographic measurements for 15 teleroentgenograms in a blinded manner. Following the reliability testing phase, all subsequent radiographic measurements were carried out by one of the authors (STO). Lower limb deformity was defined as a difference of more than 5° in mLDFA or MPTA in both lower extremities or a difference in WLL of more than 1 cm. Patients were divided into two groups, with deformity and without deformity.

### Statistical analyses

Reliability was assessed using the intraclass correlation coefficient (ICC) and 95% confidence interval (CI) within a two-way random-effect model, assuming a single measurement and absolute agreement [8]. A target ICC value of 0.9 and a 95% CI width for 0.2 for two observers were utilized to determine the minimal sample size, which was found to be 15 radiographs using Bonett’s methods [9]. 

Descriptive statistics such as means and standard deviations were used to summarize patient demographics and radiographic measurements. The Wilcoxon rank-sum and Fisher’s exact tests were used to compare variables. Univariate Firth logistic regression analysis was used to analyze the risk factors affecting the occurrence of lower extremity deformities.

All statistical analyses were performed using R version 4.0.1 (R Foundation for Statistical Computing) and RStudio version 1.3.959; (PBC). The R package ‘’logistf’’ was used for Firth’s bias-reduced logistic regression. All statistical analyses were two-tailed, and p-values < 0.05 were considered statistically significant.

## Results

### Patient demographics

After implementing the inclusion and exclusion criteria, 83 patients with solitary osteochondromas were finally included. The mean age of the patients was 12.3 ± 2.9 years. A summary of the patient demographics is presented in Table 1. Lower limb deformities were observed in 8 (9.6%) of the 83 patients. Among the 8 patients, leg length discrepancy (LLD) was observed in 3 cases, while coronal angular deformity (genu varum or genu valgum) was present in 2 cases. The remaining 3 patients exhibited both LLD and coronal angular deformities.

Excellent interobserver reliability was observed for all radiographic measurements, with ICC values of 0.969 for MPTA, 0.987 for mLDFA, and 0.958 for WLL.

**Table 1:** Summary of patient demographics

Variables	
Sex (male / female)	53 / 30
Age (years)	12.3 ± 2.9
Laterality (right / left)	37 / 46
Bone (distal femur / proximal tibia)	40 / 43
Direction (medial / lateral)	67 / 16
Type (pedunculated / sessile)	44 / 39
Distance from physis (mm)	11.9 ± 7.3
mLDFA difference (degrees)	-0.7 ± 1.3
MPTA difference (degrees)	0.1 ± 2.5
LLD (mm)	0.3 ± 3.0

mLDFA, mechanical lateral distal femoral angle; MPTA, medial proximal tibial angle; LLD, leg length discrepancy

### Patients with surgical intervention

Three patients underwent surgical intervention to correct the lower extremity deformities. Excision of the osteochondroma and guided growth to correct the deformity were performed in all 3 cases.

**Fig. 3:**
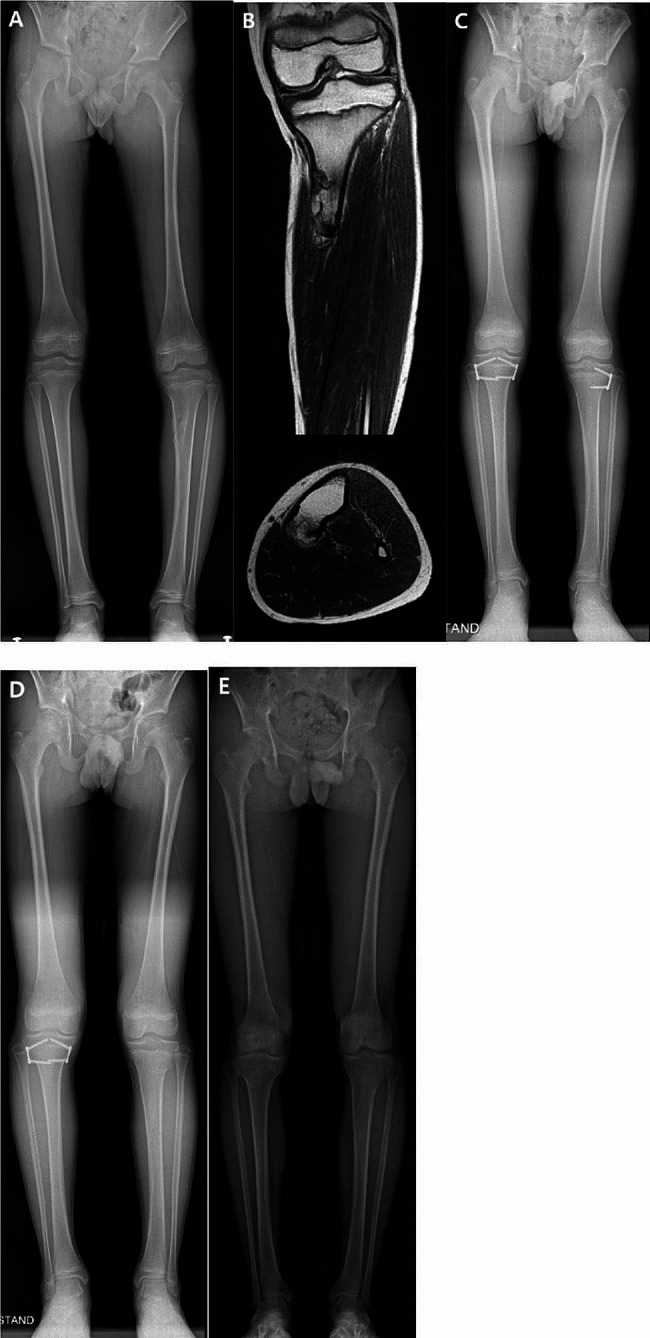
(**A**) An eleven-year-old boy with a lower limb deformity and genu varum of the left knee. (**B**) Magnetic resonance imaging showed a sessile-type osteochondroma on the posteromedial aspect of the left tibia. (**C**) Hemiepiphysiodesis for the left proximal tibia and epiphysiodesis for the right proximal tibia using a tension band plate was performed. (**D**) The tension band plate was removed after 1 year for the left proximal tibia and after 1 year and 10 months for the right proximal tibia. (**E**) The final teleroentgenogram showed the correction of LLD and coronal angular deformity

An 11-year-old boy presented to the clinic with apparent genu varum of the left knee. Radiography revealed a sessile bony mass on the posteromedial side of his left proximal tibia, associated with genu varum, with 77.1° of MPTA and 15 mm of LLD. No sagittal plane deformity was noted. A bone scan revealed a solitary mass and magnetic resonance imaging (MRI) revealed a 45 mm base sessile type osteochondroma. The patient underwent hemiepiphysiodesis of the left proximal tibia and epiphysiodesis of the right proximal tibia by using a tension-band plate. The tension band plate was removed after 1 year for the left proximal tibia and after 1 year and 10 months for the right proximal tibia. The final teleroentgenogram at the age of 15.5 years showed the resolution of the LLD and coronal angular deformity (Fig. 3).

A 12-year-old boy presented to the clinic with a palpable mass on the medial aspect of the right proximal tibia. Radiographs showed 17 mm of LLD and an 77.8° of MTPA. The MRI findings were consistent with a sessile-type osteochondroma with a 26 mm base. Excision of the mass and hemiPETS (percutaneous epiphysiodesis using transphyseal screws) for the right proximal tibia, and PETS of the left proximal tibia were performed. After a duration of 1 year 3 months, all implants were removed, resulting in the correction of LLD and genu varum deformity.

A ten-year-old boy came to our clinic with an incidental radiographic finding of a bone mass on the medial aspect of his distal femur. He had a varus deformity of the left knee with a 97.7° of mLDFA. MRI showed a 25 mm base sessile type osteochondroma. The patient underwent mass excision and hemiepiphysiodesis of the lateral aspect of the distal femur using tension band plate. The patient underwent plate removal at 9 months after the initial surgery and maintains a corrected state of genu varum at 1 year and 6 months postoperative.

The other five patients with deformities did not undergo surgery. Three patients with LLD less than 15 mm and one patient with 5 degrees of genu valgum did not require surgery. The one patient with an LLD of 28 mm and 9 degrees of genu valgum did not want surgical correction of deformity.

Among the 75 patients without deformities, the mass was surgically excised in 13 due to pain or limited motion of the joint.

### Risk factors for deformity

Comparing the variables between the two groups, there was a significant difference in the type of osteochondroma (*p* = 0.004). The osteochondromas of 8 patients with lower-extremity deformities were all sessile types. However, the differences in sex, age, laterality, bone involved, direction, and distance from the physis to the osteochondroma between the groups were not statistically significant (*p* = 0.999, 0.853, 0.999, 0.999, 0.178, and 0.165, respectively) (Table 2).

**Table 2:** Comparison of variables between patients with and without lower extremity deformity

	With deformity (*N* = 8)	Without deformity (*N* = 75)	*P*-value
Sex (male / female)	5 / 3	48 / 27	0.999
Age (years)	12.4 ± 1.8	12.1 ± 2.8	0.853
Laterality (right / left)	4 / 4	33 / 42	0.999
Bone (distal femur / proximal tibia)	4 / 4	36 / 39	0.999
Direction (medial / lateral)	5 / 3	62 / 13	0.178
Type (pedunculated / sessile)	0 / 8	44 / 31	0.002
Distance from physis (mm)	14.6 ± 7.5	11.8 ± 15.3	0.165
mLDFA difference	3.2 ± 4.2	-0.8 ± 1.2	0.073
MPTA difference	4.2 ± 3.5	0.0 ± 0.9	0.192
LLD (mm)	12.6 ± 3.4	0.6 ± 3.5	0.002

mLDFA, mechanical lateral distal femoral angle; MPTA, medial proximal tibial angle; LLD, leg length discrepancy

Because the number of deformity events was rare, the Firth logistic regression method was used to obtain an appropriate odds ratio (OR). Contrary to our initial assumptions, the distance from the physeal plate did not affect the results. The type of osteochondroma (sessile type) was the only risk factor for lower extremity deformity, with an OR of 24.0 (Table 3).

**Table 3:** Risk factors for lower extremity deformity in patients with solitary osteochondroma

Variables	Univariable Firth logistic regression		
	OR	95% CI	*P*-value
Sex (male / female)	1.1	0.24 to 4.56	0.874
Age (years)	1.0	0.88 to 1.13	0.912
Laterality (right / left)	0.8	0.19 to 3.27	0.736
Bone (distal femur / proximal tibia)	0.9	0.22 to 3.83	0.911
Direction (medial / lateral)	2.9	0.62 to 12.58	0.164
Type (pedunculated / sessile)	24.0	2.82 to 3147.31	0.001
Distance from physis (mm)	1.0	0.94 to 1.04	0.983

OR, odds ratio; CI, confidence interval

## Discussion

Little is known about deformities of the lower extremity as a consequence of solitary osteochondromas. We found that lower-limb deformities occurred in 8 of 83 patients with solitary osteochondromas. Our initial assumption was that both the tumor type and distance from the physis were risk factors for lower-limb deformities. However, the tumor type was associated with lower extremity deformities, whereas the distance to the physeal plate was not.

In previous studies of HME, the area around the knee joint was a relatively common site of osteochondromatosis. Lower limb deformities, including leg length discrepancies, genu valgum, and fixed flexion deformities, are known to be caused by osteochondromas around the knee joint [2, 10–12]. The mechanisms of deformation have been discussed in the context of HME.

Growing exostoses are thought to distort local bone growth. Porter et al. reported an inverse correlation between osteochondroma size and relative bone length in patients with HME [13]. They demonstrated the local effect of growing osteochondromas by restoring normal bone development after surgical excision of the tumor [14]. Carroll et al. demonstrated a correlation between the severity of angular deformities and the percentage of sessile lesions in HME patients [15]. Liu et al. also found that sessile lesion was significantly associated with genu valgum in 112 knees for patients with HME. [16] They postulated that more force is exerted on the underlying physis because of the increased width of the sessile osteochondroma. A broad base may exert a greater physeal effect because an osteochondroma reproduces the structure of the bone from which it originates. Thus, sessile lesions might be associated with a higher possibility for coronal limb malalignment, which was consistent with the finding of our study although the exact biomechanical effects of the broad base of tumor on the physeal plate was not clear.

On the other hand, there is a “field change” effect from a genetic mutation that distorts bone growth in HME patients. Exostosin 1 (EXT1) and Exostosin 2 (EXT2) mutations in HME result in decreased heparan sulfate levels, which are associated with ectopic bone formation. Defective biosynthesis of heparan sulfate increases proliferation rates and disrupts the differentiation process [17]. Therefore, the severity of skeletal dysplasia is correlated with the genotype, as patients with EXT1 mutations are more severely affected than those with EXT2 mutations [14, 18, 19]. 

Somatic mutations in EXT genes are sporadic in solitary osteochondromas [20]. Recent studies have demonstrated that heterozygous mutations in EXT1 are detected equally in solitary osteochondromas and HME, whereas mutations in EXT2 are infrequent in solitary osteochondromas [1]. Otherwise, there is a paucity of studies that have investigated genetic context of solitary osteochondroma. Therefore, further studies are necessary to identify the association between genetic mutations in solitary osteochondroma and lower extremity deformity.

While the literature on skeletal deformity associated with HME is extensive, there is a paucity of studies related to solitary osteochondroma and lower limb deformity. In 2008, Florez et al. reported that one patient had valgus knee deformity associated with a solitary mass in the proximal tibia among 113 cases of solitary osteochondroma. [4] Recently, Park et al. retrospectively reviewed 111 patients with solitary osteochondroma around the knee and found that it did not cause a clinically significant deformity of the lower extremity. However, they concluded that solitary osteochondroma in the distal femur was associated with shortening of the affected limb. [21] In our study, we demonstrated that solitary osteochondroma around the knee, especially the sessile type, was associated with lower extremity deformity.

This study had some limitations. First, a cross-sectional design was employed. Even if patients did not have lower extremity deformities at the time of the study, it is possible for them to develop deformities as their growth continues. Given that studies have shown that HME deformities become severe as skeletal maturation progresses, a long-term prospective study would be more informative. Second, there were insufficient data to perform statistical validation. We performed a univariate logistic regression analysis to analyze the risk factors instead of a multivariate analysis because of the insufficient number of deformities. The wide confidence interval for the ORs of risk factors might also be due to limited data. Therefore, further studies with larger cohorts are required to identify sophisticated risk factors for lower extremity deformity in patients with solitary osteochondromas. Third, the local biomechanical properties of the tumor, such as the size or width of the solitary osteochondroma, may also be a risk factor for deformity. As MRI is required to measure the accurate size of the tumor, we could not measure the tumor size or width because of the absence of MRI data for all patients. It is desirable that this dimension is included in future studies.

## Conclusion

In our cohort with solitary osteochondroma, lower limb deformities were observed in 8 (9.6%) out of the 83 patients and these were significantly associated with sessile-type tumors. Therefore, patients with sessile-type solitary osteochondroma around the knee require careful surveillance of lower limb alignment with whole leg teleroentgenoram.

## Data Availability

The data set supporting the conclusion of this article is available on request to the corresponding author.

## Abbreviations

HME: hereditary multiple exostoses
mLDFA: mechanical lateral distal femoral angle
MPTA: medial proximal tibial angle
WLL: whole limb length
ICC: intraclass correlation coefficient
LLD: leg length discrepancy
MRI: magnetic resonance imaging
